# Rational Protein Engineering Guided by Deep Mutational Scanning

**DOI:** 10.3390/ijms160923094

**Published:** 2015-09-23

**Authors:** HyeonSeok Shin, Byung-Kwan Cho

**Affiliations:** Department of Biological Sciences and KI for the BioCentury, Korea Advanced Institute of Science and Technology (KAIST), Daejeon 305-701, Korea; E-Mail: ckckck229@kaist.ac.kr

**Keywords:** deep mutational scanning, next generation sequencing, high-throughput screening, protein engineering

## Abstract

Sequence–function relationship in a protein is commonly determined by the three-dimensional protein structure followed by various biochemical experiments. However, with the explosive increase in the number of genome sequences, facilitated by recent advances in sequencing technology, the gap between protein sequences available and three-dimensional structures is rapidly widening. A recently developed method termed deep mutational scanning explores the functional phenotype of thousands of mutants via massive sequencing. Coupled with a highly efficient screening system, this approach assesses the phenotypic changes made by the substitution of each amino acid sequence that constitutes a protein. Such an informational resource provides the functional role of each amino acid sequence, thereby providing sufficient rationale for selecting target residues for protein engineering. Here, we discuss the current applications of deep mutational scanning and consider experimental design.

## 1. Protein Engineering in the Ultrahigh-Throughput Sequencing (uHTS) Era

Protein engineering has extensively been applied to several fields of biotechnology, including the medical sciences and for industrial applications. Since the first report of protein engineering by site-directed mutagenesis in 1982, numerous proteins have been engineered using various techniques developed by technological advances [1]. Advances in protein structure determination techniques have had a great impact on protein behavior prediction and mechanisms, which were integrated with the rational strategy of protein engineering as an informational resource [2,3,4]. Such protein structure data have allowed the developments of modeling methods such as Rosetta and RosettaDesign, which enables the prediction of protein structure for novel as well as engineered proteins [5,6]. Improved computational and modeling power in addition to accumulation of protein structure and mechanism information have introduced a semi-rational method of protein engineering that uses predictive algorithms to preselect potential target sites [7,8]. Developments in high-throughput screening systems have had a great impact on the directed evolution strategy of protein engineering. Developments in application of high-throughput screening methods such as enzyme-linked immunosorbent assay and flow cytometers, such as fluorescence activated cell sorting (FACS), have reduced the time and effort involved in protein engineering [9,10]. Thus far, protein engineering, as a field, has grown along with development of other biotechnologies and it is only natural that recent advances in high-throughput sequencing are applied to protein engineering [11].

Integration of high-throughput sequencing technology with protein engineering involves the coupling of protein variant generation with a high-throughput screening system. Instead of selecting a few selected protein variants, high-throughput sequencing allows the sequencing of millions of protein variants, termed as deep mutational scanning [11]. Depending on the selection pressure, both positive and negative phenotypes by protein mutations can be linked to the sequence space [12]. Such an information load with regard to the protein sequence space has a great impact on the field because it solves some of the problems that occur as a part of current protein engineering strategies. For rational and semi-rational strategies of protein engineering, extensive information on protein structure is required for selecting a target site for engineering. Furthermore, such a selection or pre-selection algorithm shows difficulty in prediction of sites that are distant from binding or active sites [13,14]. On the other hand, directed evolution is another protein engineering strategy comprising two-steps: (1) mutagenesis to generate the mutant library of the protein and (2) screening for the protein variant with the desirable property. For example, phage display has been used to display proteins or peptides on the phage surface and, when followed by affinity captures, allows selection of proteins with desired properties without much prior knowledge on the protein structure or mechanism of function [15,16,17]. Thus, structural or functional information of a protein is not necessary, provided that selection pressure or screening method for desired phenotype is available [18]. However, this approach can only screen for a limited number of engineered products compared to the millions of possible mutations that are contained in the initial mutant library due to the requirement for DNA sequencing. From this perspective, deep mutational scanning provides the required depth for sequencing millions of possible mutations, which, in turn, provides information on the important sites, which is not necessarily when the distance to the known active sites is close. Furthermore, in contrast to the conventional method of directed evolution, where iterative mutagenesis and screening are performed until the desired phenotype is found, the screening system used in deep mutational scanning can be as simple as function on/off that saves time and effort.

## 2. Deep Mutational Scanning

### 2.1. Overview

Deep mutational scanning as a concept is quite exciting. It identifies the effect of all possible amino acid changes for each position in a protein and compares the enrichment of mutation to appropriate selection pressure depending on the characteristics of the protein. This is enabled by the delivery of the information between thousands or millions of mutations to protein variants’ phenotype by high-throughput sequencing. Compared to the initial protein variant library, the mutations quantified after selection enable identification of the effect of mutations in certain residues to the functional change of the protein variant. Overall, the elucidation of such vast information on protein sequence space in relation to selection pressure aims to determine the protein sequence and function relationship [11]. In addition, the analysis of the mutations in the protein variants reveals a sequence–structure–function relationship, which is difficult to predict [19,20]. In terms of strategy, deep mutational scanning uses the discovery-based protein engineering methods to determine the protein sequence and function relationship by linking the mutational genotype to the phenotype, which then provides the basis for hypothesis-driven protein engineering.

Because deep mutational scanning is a relatively new concept that differs from conventional protein engineering strategies and experimental designs, this review introduces the basic overview of the methodologies that help determine the experimental design as well as the computational analysis using recent studies. Precautions for data interpretation are also discussed along with perspectives on the current limitations and future developments.

**Figure 1:**
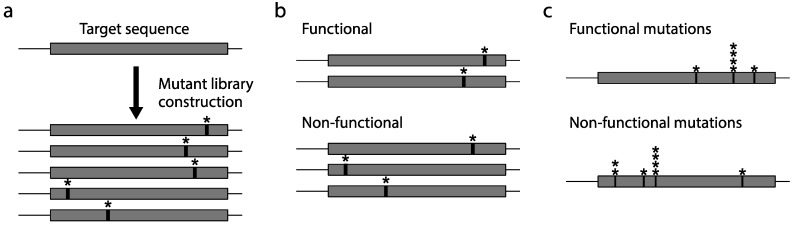
Schematics of the simplified overview of deep mutational scanning: (**a**) generation of the initial protein variant library for target protein sequences; (**b**) screening for protein variants with desired properties; and (**c**) sequencing and quantification of the mutations under different selection pressures. The asterisks indicate mutations at a specific site and the stacked asterisks indicate enrichment of mutations in specific sites after quantification. For example, mutation counts at different sites are shown with * positions carrying a mutation, ** positions carrying two mutations, and **** positions carrying four mutations.

To design a deep mutational scanning experiment for a target protein, three conceptual steps that involve construction of the protein variant library, screening, or selection for functions, and high-throughput sequencing must be considered. First, a library containing the mutated sequence of the target protein is generated, which is used as the input variant library (Figure 1a); Second, the input variant library is expressed and subjected to a screening system that can concordantly link the sequence variant to the functional variant (Figure 1b); Third, the selected protein variants are subjected to high-throughput sequencing and the mutation spectra in different libraries are quantified (Figure 1c). Although Figure 1 illustrates the simplified overview of the processes involved in deep mutational scanning, different methodologies and schemes may be applied. The mutational library can be generated by random mutagenesis, saturated mutagenesis, or DNA synthesis. The selection pressure in deep mutational scanning depends on the protein’s characteristic and the experimental design. It can be applied to assays of binding affinity, enzymatic assays, as well as fluorescence signals [21,22,23,24].

### 2.2. Mutagenesis

The desired sequence space and the size of the protein must be considered for the initial mutant library generation. For instance, a hypothetical protein “hyp-A” constituted by 100 amino acids would require the generation of 1900 different variants for single site mutagenesis of all residues that constitute the protein. To obtain an unbiased mutant library that constitutes all possible sequence space requires synthesis of 1900 oligonucleotides or 3800 primers. The complete coverage of initial library over the sequence space and unbiased concentration of each mutant is important because the initial library is also sequenced as a control [22]. To validate that a mutation site in a protein is important because it has been enriched after selection, the initial library data must show that the mutation was evenly distributed. Thus, a protein variant library generation method such as single-site saturation mutagenesis (S-SSM) is very effective in terms of the completeness and uniformity, as all 20 possibilities of amino acid changes for each residue is designed [23,24,25,26].

Oligonucleotide-directed random mutagenesis is another mutagenesis method frequently used in deep mutational scanning [10,22,27,28,29,30,31,32,33,34,35,36]. This method uses degenerative oligonucleotides that contain the “NNS” or “NNK” codon, where N represents any of the four-nucleotide sequences, S represents either G or C and K represents T or G [10,30]. This method is effective in that all sequence spaces of the target protein can be theoretically covered with one order of oligonucleotide synthesis compared to S-SSM, where 19 oligonucleotide are required [37]. However, there is also a possibility that not all sequence spaces maybe covered in the initial library. As oligonucleotides are randomly synthesized, the possibility of an amino acid being generated from the random combination of NNS or NNK is not equal. There is a chance that the generated NNS sequence may not be evenly distributed and that some of the mutations may be lost in the transformation or expression system. To ensure the complete sequence space of all amino acid variants are covered, the depth required for screening must be carefully calculated. From a practical viewpoint, oligonucleotide-directed random mutagenesis is cost efficient but there is a possibility of some mutations being lost from the initial library. Therefore, while S-SSM is relatively cost-intensive and time consuming, the generated library would be complete and unbiased.

Another method for generating a protein variant library is random mutagenesis by PCR [21,38,39,40,41]. This method can be considered for target proteins or sequences that too are long to use oligonucleotide-directed method, as it is the most efficient in terms of cost and time. However, PCR-based mutagenesis does not cover all possible sequence spaces. Depending on the initial nucleotide of a codon, only 12 mutations out of 19 are possible unless a consecutive mutation is introduced in the codon. Furthermore, random mutagenesis by PCR is known to have some bias in the mutation spectra. Depending on the mutation frequency and the coverage of the mutant library, such a bias may be negligible, as less biased PCR mutagenesis has been developed [42]. For example, Shin *et al.* used PCR-based random mutagenesis, which showed that the mutation frequency of AT-to-NN was similar to GC-to-NN [21]. Overall, each method for generating the initial mutation library has its advantages and disadvantages and the best fitting method may differ from case to case. Furthermore, although this review followed the sequential steps of experiment, the method to generate the initial mutant library should consider all aspects of the process such as the expression system, selection pressure, and sequencing methods.

### 2.3. Construction of a Protein Variant Library

**Figure 2:**
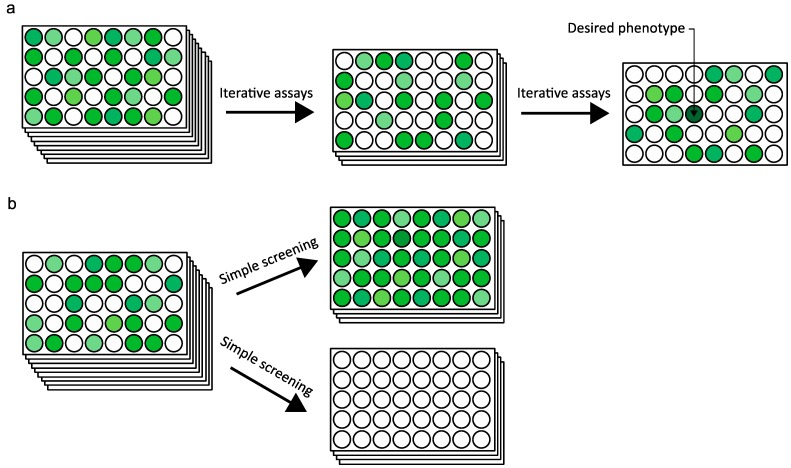
Schematics of the simplified screening systems of the (**a**) conventional strategy of directed evolution, where iterative assays are performed until a desired phenotype appears; and (**b**) deep mutational scanning, where the protein variants are screened to a simpler selection pressure. The different phenotypes of the protein variants are shown by gradient of green colored wells. The desirable phenotypes are shown by darker green colored wells and loss of function is shown by white colored wells.

The expression system for deep mutational scanning is not too different from the conventional protein engineering methods that require a selection system. Similar to any directed evolution strategy in protein engineering, the expression system requires a link between the mutated DNA and the protein variant that enables determination of the corresponding mutation responsible for the changed phenotype [43]. On the contrary, the selection pressure for deep mutational scanning is quite different from that in conventional protein engineering. A directed evolution strategy of protein engineering involves performing repeated screening assays until the desired phenotype is found (Figure 2a). Such repeated assaying is costly, time consuming, and laborious since there is no guarantee that the desired phenotype will appear. On the other hand, deep mutational scanning uses a different strategy in screening the protein variants (Figure 2b). Instead of performing iterative assays to find the protein variants with the desired phenotype, a simple screening pressure such as function on/off or binding affinity is used to divide the selected and unselected protein variants. The massive amount of information from loss of function variants is also analyzed to find sites critical to the mutation or to protein fitness [44].

Recent studies have shown that three types of expression systems are frequently used in deep mutational scanning—the plasmid system, phage or yeast display system, and bacterial or yeast two-hybrid system (Table 1). Among these expression systems, the phage display system is used for the investigation of proteins known for their protein–peptide and protein–DNA binding interactions, where assays using binding affinity are favorable [45]. For example, Fowler *et al*. used the phage display system to investigate the affinity of binding of the WW domain (named by the two conserved tryptophan residues in the domain) to its peptide ligand [22]. Using beads with the peptide ligands attached, six successive rounds of selection and washing were performed, which allowed the variant WW domain with better affinity to be enriched. The advantage of this display system is that both the protein and the encapsulated DNA are easily accessible. As the protein is displayed in the exterior part of the phage, the use of cytoplasmic phages such as T7 allows efficient delivery of the proteins [46]. Thus, the phage display system is an effective combination that can be used in functional protein binding assays in deep mutational scanning.

**Table 1:** Recent studies using deep mutational scanning.

Mutation Generation Method ^1^	Variant Library	Sequencing Method ^2^	Target Protein ^3^	Reference
ORM	Phage display	Solexa/PE	PSD95^pdz3^	[10]
ORM	Bacterial two-hybrid	Illumina/PE	hYAP65	[22]
ORM	Yeast two-hybrid	Illumina/SE	BRCA1	[27]
PRM	Plasmid	Illumina/SE	EcFbFP	[21]
SM	Yeast display	Illumina/PE	HB80.3	[24]
ORM	Plasmid	Illumina/PE	APH(3′)II	[28]
SM	Plasmid	Illumina/PE	Bgl3	[23]
SM	Plasmid	454	CcdB	[26]
ORM	Plasmid	Illumina/PE	Pab1	[29]
ORM	Mammalian display vectors	454	IgG	[30]
ORM	Ribosome display	454	CDR loops of Fab	[47]
ORM	Phage display	Illumina/PE	hYAP65	[48]

^1^ ORM: Oligonucleotide-directed random mutagenesis; PRM: PCR-based random mutagenesis; SM: Saturated mutagenesis; ^2^ PE: paired-end; SE: single-end; ^3^ PDZ domain: post synaptic density protein; hYAP65: human Yes-associated protein 65; BRCA1: breast cancer 1 (early onset); EcFbFP: *Escherichia coli* flavin mononucleotide binding fluorescent protein; HB80.3: HB80.3 (designed high affinity binding protein); APH(3′)II: Tn5 transposon derived aminoglycoside-3′-phosphotransferase-II; Bgl3: β-glucosidase; CcdB: bacterial toxin protein CcdB; Pab1: poly(A)-binding protein; IgG: immunoglobulin G; CDR: complementary determining region; Fab: fragment antigen-binding region.

The “two-hybrid” system is a screening system used in protein–protein and protein–DNA interactions to activate a downstream gene by binding it to a transcription factor [49,50]. One of the advantages of this system lies in the capability to quantify the downstream reporter genes *in vivo*. McLaughlin *et al.* have used the bacterial two-hybrid system to quantitatively link the expression of fluorescent proteins, which enabled FACS to be used as the selection system [10]. Using this system, the authors found nine evolutionarily important sites in PSD05^pdz3^, which were tested by repeated experiments with different peptide ligands. This study is exemplary in that the discovery-based approach was used to gain information and the gained information was used to successfully apply hypothesis-driven engineering. The two-hybrid system is very effective and efficient in that the reporter system allows rapid quantitative analysis. Although this system requires the preparation of the two-hybrid system for either bacteria or yeast, this system would be a very good candidate for deep mutational scanning in proteins with binding affinity functions.

The plasmid expression system is one of the oldest protein expression systems that is relatively easy to handle [51]. For applications in deep mutational scanning, the plasmid system is favorable for proteins with catalytic activity that require *in vivo* cell-based screening assays [23,26,28,29]. Unlike the phage display or the two-hybrid method, where the protein’s ligand binding affinity and interaction is measured, proteins with any function can be assayed using the plasmid system. However, besides the advantage of the high degree of freedom in terms of target protein choice, this system does not have any particular advantages in specific screening assays. Thus, the screening system for the protein of choice must be carefully considered for application in deep mutational scanning. For example, the use of a high copy plasmid may cause an overflow of proteins, which would hamper the sensitivity of the screening system; moreover, a saturation point must be measured beforehand.

### 2.4. Ultra High-Throughput Sequencing (uHTS)

**Figure 3:**
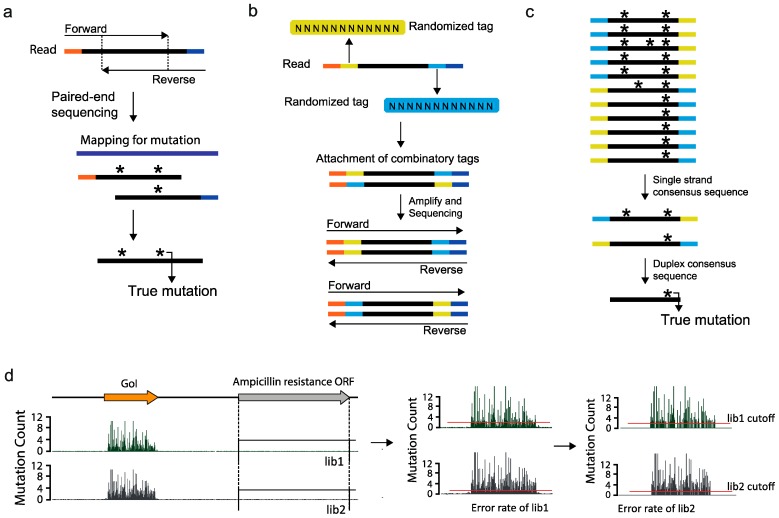
Methods to rectify sequencing errors: (**a**) a scheme of how paired end reads with short sequencing reads allow the detection of sequencing errors; (**b**) schematic showing the concept of the duplex sequencing method; (**c**) how the consensus sequence is used to remove sequencing errors, adapted by permission from the Macmillan Publishers Ltd: *Nature Protocols* [61], copyright 2014. The black bar indicate the target inserts reads for sequencing and the orange and dark blue colored bars at end of the insert reads indicate sequencing adaptors; the yellow and light blue bars indicate the randomized duplex tags; and (**d**) Hypothetical mapping of the mutation frequency for variant library sequencing. The red line indicates the sequencing error rate of the ampicillin gene used as the cutoff. The asterisks indicate mutations and the orange and blue bars at the ends of the reads indicate the sequencing adaptors.

High-throughput sequencing has generated a large amount of sequence data in different applications. In particular, the sequencing of genomes, transcriptomes, ribosome-interacting RNAs, and protein-interacting DNAs has been widely used in countless studies, regardless of the organism or the application [52,53,54]. To apply high-throughput sequencing to protein engineering, a few aspects of the sequencing process must be considered for appropriate data generation and analysis. Among these, sequencing error is a critical aspect that may hamper the accurate interpretation of the sequencing data. For example, the Illumina Genome Analyzer IIX (GA IIX), which has been used in several studies of deep mutational scanning, is known to have an approximate 0.5%–1% error rate [22,48,55]. The hypothetical hyp-A protein-encoding DNA sequence is 300-bp long, which means that the sequencing result may have three errors that are undistinguishable from the actual mutation. To this end, many methods have been developed to reduce errors in the high-throughput sequencing. For example, Lou *et al*. developed a circle sequencing method that involves denaturing DNA into single-stranded DNA (ssDNA) followed by circularization [56]. By using random primers and Phi29 polymerase that continuously replicate around the ligated circle, many amplified copies of the reads are generated and sequenced to find the consensus sequence with true mutation [57]. Another method to distinguish sequencing error from the true mutation is to use paired-end sequencing with a short read length, which will result in the formation of an overlapping region between the forward and the reverse paired reads. This overlapping region enables the identification of the true mutation, thus significantly reducing the error rate (Figure 3a). Other methods developed to reduce sequencing error rates use tags or barcodes in the sequencing library construction step to find the consensus of sequenced reads [58,59,60]. For example, the duplex sequencing method is known to enable detection of very low frequency mutations by using randomized barcodes at both ends of a read [61,62]. To detect very low frequency mutations, this method enables the detection of both sequencing and PCR errors that result from the construction of the sequencing library. The sequencing library is constructed using the randomized duplex tags of 11 bp downstream and upstream of the sequence, which allows all the reads to have a unique barcode. The reads are then amplified in both the forward and backward manner, which results in at least two sequence reads with the reversed barcode (Figure 3b). After sequencing, the consensus of each of the sequence reads is obtained for the reads with the same barcode (Figure 3c). By comparing the forward and the backward sequences of the reads, the potential errors generated from PCR are eliminated. Finally, comparing the single strand consensus of the same barcodes, but in different directions, allows the detection of the true mutation. Thus, the use of this method can minimize the error rate to a minimum, which would be very effective for deep mutational scanning experiments that require highly sensitive quantification of the mutation.

In addition to the sequencing library generation methods to reduce sequencing and PCR error rate, extra precaution can be taken to further remove any false positives during the quantification of the mutation. For example, Shin *et al.* used a plasmid system that had an ampicillin resistance gene [21]. Since the *E. coli* would not survive in the presence of ampicillin without the resistance gene, mutations that had a lower frequency than the sequencing error rate found in the ampicillin-resistance gene were removed from the gene of interest (GoI) (Figure 3d). In this case, some coverage of the possible sequence space would be lost as a result of the loss of the low frequency mutation. However, the primary interest in deep mutational scanning is directed towards the enriched mutational sites, and a cutoff of the mutation frequency would not affect the major sites. Thus, for mutational scanning system results that are expected to show a high frequency for mutations in iterative cycles, a cutoff for mutation frequencies might help in downstream analysis.

Other aspects of the sequencing process that must be considered are the sequencing depth and library diversity. The sequencing depth can be calculated by considering the length of the target protein and the number of possible variants. For example, hyp-A is 300 bp long and the number of possible variants is 1900. By multiplying the protein length with the number of possible variants, we find that 570,000 is the number of base pairs required for the sequencing depth to be 1×. Thus, sequencing of two variant libraries with 100× depth requires 114 Mb, which is not a problem considering that the current benchtop sequencing platforms can produce gigabytes of data [63]. However, sequencing of very short sequences means that the sequence diversity would be very low, which is known to cause sequencing errors. For an Illumina platform, sequencing is performed by imaging the color produced by the clusters of the platform, and when the diversity is low, the color intensity cannot be properly detected, which causes errors in sequencing [64]. The easiest way to avoid sequencing errors is by increasing the spike-in percentage of the PhiX, which may complicate the calculation of the sequencing output. In addition, an extra sequence containing a series of random N sequences can be attached upstream of the insert reads to solve the low diversity problem. For now, it appears that the best sequencing pipeline for deep mutational scanning is MiSeq (Illumina), which offers numerous cartridges for different read lengths (50, 150 and 250 bp) with paired-end capability and contains a Real Time Analysis (RTA) software known to improve the data quality of low diversity samples [65].

### 2.5. Data Interpretation

As is the case for all applications of high-throughput sequencing data, the data analysis requires to be coupled to a logically and statistically acceptable workflow to answer the biological question or determine its relevance. For deep mutational scanning, the protein’s sequence and function relationship is the main question towards which the data analysis should be directed. Conceptually, this can be divided into two parts: analysis of the data to generate a mutational map or table and data interpretation of the mutational map. While data analysis involves bioinformatics to determine the abundance of each mutation at all positions, data interpretation is directed at determining the biological implication by calculating the abundance of mutations to different amino acids at different sites.

**Figure 4:**
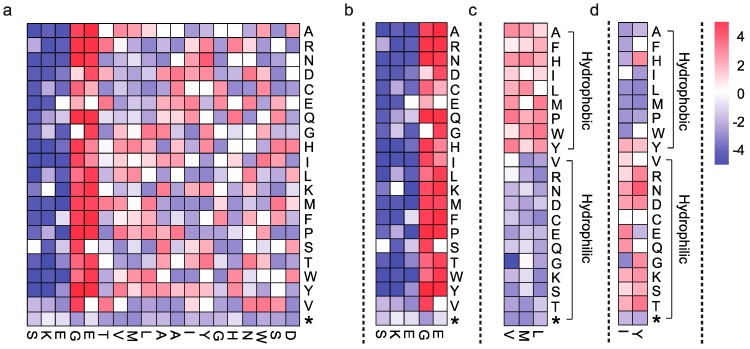
(**a**) A hypothetical mutational map generated to show mutation frequency at each position; Part of the mutational map showing (**b**) extremely tolerant and critical residues to mutations; (**c**) tolerant to hydrophobic mutations and (**d**) tolerant to hydrophilic mutations. The x-axis indicates the protein residues and the y-axis indicates the possible amino acids. The color key represents the mutation frequency at each amino acid. The white color indicates that no mutation was found. The blue color indicates mutation frequency of loss-of-function variants and red color indicates mutation frequency of function-retained variants. The stop codon is indicated by *.

The nature of deep mutational scanning data is that it contains many mutations. Thus, data analysis is directed at identifying the mutations that result in a codon change in genes coding for different amino acids and determining the abundance of such mutations at all positions for each amino acid. The first part of deep mutational scanning data analysis does not differ from the conventional treatment of sequencing data. After removal of low-quality reads, the data should be mapped to the vector system sequence, the organism genome, and PhiX and only the unmapped reads should be collected. This step essentially removes any possible contaminations. If the sequencing library is constructed with the sequencing error precautions described in Figure 3a,b, the sequencing error can be removed by scripts called Enrich and by several scripts provided by Kennedy *et al*., respectively [61,66]. After the reads are mapped to the reference sequence, which in this case would be a protein, the mapped reads are extracted by SAMtools, and each read is translated into an amino acid [21,67]. The translated amino acids are then compared to the reference amino acid sequences to define mutations, and the abundance of these mutations is determined. As an extra precautionary measure, in a variant library that is expected to contain only single-site mutations, reads with more than one amino acid should be disregarded. Thus, the basic workflow of the deep mutational scanning data analysis is that the reads need to be translated into the corresponding amino acids first and then compared to the reference to identify and quantify mutations. The downstream analysis for data interpretation is then processed from this mutational table or map, which contains the abundance of each mutation (Figure 4a). It is also notable that there are tools that have been designed specifically for the analysis and visualization of deep mutational scanning data, which are Enrich and dms_tools [66,68].

Interpretation of deep mutational sequencing data starts from the investigation of sites that have enriched mutations. With this perspective, it is advantageous to generate a colored heatmap from the data in the mutational table because it contains information that is easier to interpret by visualization (Figure 4a). First, the sites that are highly tolerant to mutations and those critical to mutations can be identified (Figure 4b). The sites with mutations indicated in blue color are enriched with every amino acid change, suggesting that these sites are critical to protein function, *i.e*., these sequences have a structurally or functionally important characteristic. Sites with mutations indicated in red color are bombarded with every amino acid change, suggesting that these sites are tolerant to mutations, *i.e*., these residues do not affect protein function or that their effects are negligible. This information is important because it identifies structurally important residues that are sequentially and structurally far from the active sites and difficult to predict. Sites with enriched mutations after selection are also quantified by different equations in different studies to show the importance of these residues. The characteristics of the residues can also be identified by investigating the type of amino acid changes. For example, the heatmap in Figure 4c,d is generated with the same dataset as that of the heatmap in Figure 4a (residues 7 to 9 and residues 12 to 13), but is generated with a different order of amino acid changes. From these hypothetical data, it can be inferred that the hydrophobic and hydrophilic characteristic of these residues greatly affects protein function (Figure 4c,d). Residues with a strong hydrophobic characteristic could be a site of dimer formation or a hydrophobic substrate-ligand pocket [21,69]. Such information can be used to define the protein stability in different environment as the function of the protein is closely related to the structural integrity of the protein. The results of the data interpretation are good examples of how the wealth of information generated from deep mutational scanning is used.

### 2.6. Limitations and Future Perspectives

The advance of high-throughput sequencing technology and screening assays has led to the development a new strategy of protein engineering called deep mutational scanning. This strategy has succeeded in showing the mutational consequence of different proteins by using different combinations of methods for variant protein generation, screening, and sequencing. However, mutational scanning is not applicable to all proteins because not all proteins are coupled with high-throughput screening assays to investigate their function. Furthermore, analysis of multi-site mutations and their epistasis is difficult because it requires a method that can screen tens of millions of mutants and be applicable only to small-sized proteins because of the sequencing read length.

The availability of a high-throughput screening system is a difficult concern to be addressed. For deep mutational scanning, the screening system requires the DNA to be linked to the phenotype and the screening system needs to be a high-throughput system. Designing such a screening system is difficult for enzymes and proteins for which the interacting substrate is not known, especially if the protein only functions *in vivo*. Nevertheless, fashioning a screening system is a challenging task but is essential to deep mutational scanning, which is its biggest limitation. A means to overcome this problem would greatly enhance the future prospects of deep mutational scanning.

Analysis of multi-site mutations and their epistasis has been performed in a few studies [10,22,33,48,70]. The results from deep mutational scanning on a WW domain have been used to produce a model for mutational epistasis, which showed a good prediction rate of 70% [22]. Additionally, epistasis in a few mutation sites has been tested for differential ligand binding of PSD95^pdz3^ [10]. Interestingly, some mutation sites that had a deleterious effect on single-site mutagenesis showed significant epistasis to enhance binding to a different ligand [10]. For large-sized proteins, the sequencing length is insufficient to detect the effect of multi-site mutation in one read. To resolve this issue, tag-directed assembly methods can be applied [71,72,73,74]. These methods require specific barcode for each protein variants to assemble the whole protein sequence with same barcoded reads. However, unlike the random barcode system used to reduce sequencing errors this method require specific barcodes for millions protein variants. This is cost inefficient, as the sequencing library of each protein variant needs to be constructed separately. Another approach to address this problem is to analyze the single-site mutagenesis library first and select a few domains to perform another single-site mutagenesis on the selected mutants. This would require a barcode for the second single-site mutagenesis to distinguish these mutations from those of the first and second round of mutagenesis, and the sequence space will not be highly covered. In addition, the distance between the mutated domains in the first and second rounds needs to be less than the sequencing read length. Although many limitations exist, deep mutational scanning application is the most promising method to study epistasis in different mutation sites.

Overall, deep mutational scanning strategies provide mutational data of a protein by linking mutation to the phenotype. Such mutational data serve as valuable information to understand the proteins’ functional mechanism, which can then be applied to clinical application of antibodies, studies on human genetic diseases, structural protein sciences, and protein engineering [14,23,27,53]. In addition, further developments of this approach are promising because it is based on two rapidly evolving technologies: high-throughput screening and high-throughput sequencing. In this review, we have introduced different methodologies and concepts that can be applied to deep mutational scanning based on recent studies. Although some questions remain regarding the mutational epistasis of large-sized protein targets, deep mutation scanning provides answers to many questions that involve the protein’s sequence and function relationship.
